# Selective Resonance Photoionization of Odd Mass Zirconium Isotopes Towards Efficient Separation of Radioactive Waste

**DOI:** 10.1038/s41598-018-38423-4

**Published:** 2019-02-11

**Authors:** Takashige Fujiwara, Tohru Kobayashi, Katsumi Midorikawa

**Affiliations:** Attosecond Science Research Team, RIKEN Center for Advanced Photonics, 2-1 Hirosawa, Wako Saitama, 351-0198 Japan

## Abstract

We achieve a considerable improvement in proposed schemes for the selective photoionization of odd mass zirconium isotopes. The technique implements intermediate-state alignment for isotope-selective laser excitation by broadband pulsed lasers, which incorporates the spectroscopic selection rules for the absorption of polarized light. The improvement includes newly found intermediate levels, where *J* = 0 character as a third excited-state intermediate, in cooperation with four-step photoexcitation (*J* = 2–1–1–0 scheme). Isotope selectivity (separation coefficient for ^91^Zr: >2400) has been identified in addition to a significant enhancement of ionization efficiency (30×) compared with previous research. A search for suitable third intermediate levels has covered over autoionizing Rydberg states in a singly ionized Zr II region (up to 58 000 cm^−1^). The measured autoionizing Rydberg states show high photoion yields but are not identified as favoured isotopic selectivity, as observed in the four-step photoionization. Prospects and future directions in laser even/odd-mass isotope separation are discussed.

## Introduction

Isotope separation methods are crucial to numerous scientific and industrial processes. Nuclear power plants, for example, require fuels that are enriched in select isotopes and separate radioactive isotopes for disposal. The odd-mass zirconium isotopes (93 and 95) are among fission products that are radioactive. Notably, the ^93^Zr isotope reveals a long half-life of 1.5 million years, which is one of the seven major long-lived fission products (LLFPs). Separation of these highly radioactive isotopes from non-radioactive ones makes a pivotal contribution to the disposal of wastes and recycling. A laser technique may have an important role in reducing a significant amount of high-level radioactive wastes as a pre-treatment prior to undergoing nuclear transmutation processes that influence nuclear reactions and eliminate radioactivity.

In past decades, an atomic vapour laser isotope separation (AVLIS) method has been successfully proposed and employed for other major isotopes^1–3^, such as the uranium isotope ^235^U. In the AVLIS technique, the target metal is heated to ~2000 K and vapourized to form atomic vapour beams. The laser is tuned to a particular atomic transition and preferentially excites the isotope. So-generated cations (*e.g*.^235^U^+^) are separated by electric fields from the neutrals and then collected. If the spectral shifts between the target and other isotopes are larger than the bandwidth of the laser, the isotopic selectivity is reasonably high. Very recent developments of the atomic laser ionization methods achieved a high efficiency combined with a high spectral resolution, which was demonstrated on-line in a supersonic gas jet^4,5^. However, ^91^Zr is relatively difficult since it shows small optical isotope-shifts (100–200 MHz)^6–8^, and a considerably narrow-band laser needs to be tuned to a specific group of hyperfine transitions that is slightly shifted from the even mass isotope transitions. In addition, Doppler line broadening, which is associated with vapourization temperatures (>2200 K)^9^, must be diminished to improve its selectivity, and it often utilizes supersonic expansion of vapour through a nozzle^10–12^. Furthermore, to date, knowledge is lacking about the highly radioactive ^93,95^Zr isotope hyperfine structures, which hinders practical implementation.

In this study, we employed an alternative approach using intermediate-state alignment for the isotope-selective laser excitation of atoms, which exploits the angular momentum selection rules for the absorption of polarized light^13^. Linearly polarized, pulsed dye lasers are used to prepare aligned states from which further excitation of the even mass isotopes is prohibited by selection rules. Hyperfine interactions in the odd mass isotopes (^91,93,95^Zr) produce population redistributions upon photoexcitation, which causes selective excitation followed by ionization of these odd mass isotopes^14^. The method does not require the use of narrow-band lasers, does not demand a particular pulse shape or duration, and is applicable to the use of thermally distributed atomic vapours as a beam source.

**Figure 1:**
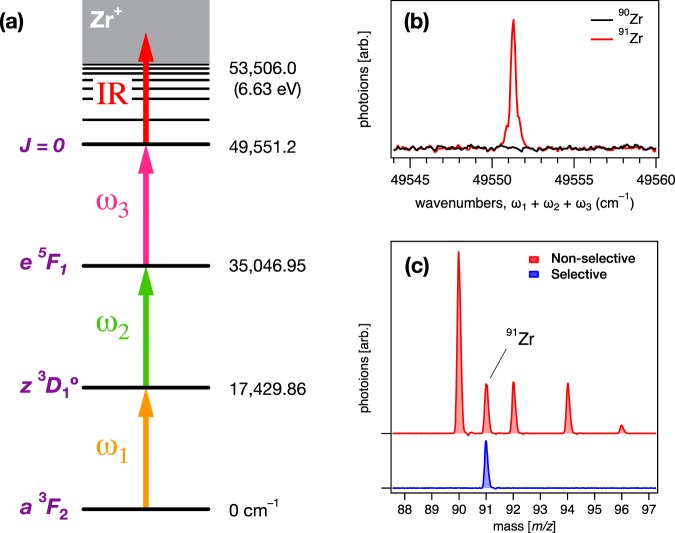
(**a**) Energy levels and transition diagram of the excitation path (*J* = 2–1–1–0 excitation scheme). Spectroscopic terms and energies are present for each level. (**b**) The mass-resolved, resonant photoionization spectra of Zr. A newly found 3rd intermediate state, with *J* = 0 character, shows a high ionization efficiency and high isotopic selectivity. (**c**) The mass spectra of the naturally occurring Zr sample obtained in the two different configurations; In the selective condition, an exclusively isotopic selectivity is observed for ^91^Zr, where all laser polarizations of *ω*_1_, *ω*_2_, and *ω*_3_ are parallel. In the non-selective condition, each isotope appears in the perpendicular polarization of *ω*_3_ to *ω*_2_ and retain the same polarizations at *ω*_1_ and *ω*_2_. The resultant separation coefficient (>2400) for ^91^Zr over the even mass isotopes is obtained. (Refer to the Theory section).

In this paper, we present basic research on the selective photoionization of the odd mass Zr isotopes with multiple and stepwise laser excitations. Accompanied by the foregoing study of the laser isotope separation of palladium isotopes^15^, we revisited the three-step resonant photoionization of Zr vapour and examined the isotopic selectivity for ^91^Zr from other naturally occurring even mass isotopes. The representative excitation scheme employed in this investigation is illustrated in Fig. 1(a–c), which demonstrates higher isotopic selectivity and ionization efficiency than previously reported data^14,16^. A comprehensive search for new intermediate states, including autoionizing Rydberg states above the ionization potential of Zr I, have been conducted. To enhance the isotopic selectivity, we discuss important parameters, such as intermediate state alignments with laser light, input laser fluence on each state, and selective ion yields in multi-step photoionization.

## Theory of Selective Photoexcitation

**Figure 2:**
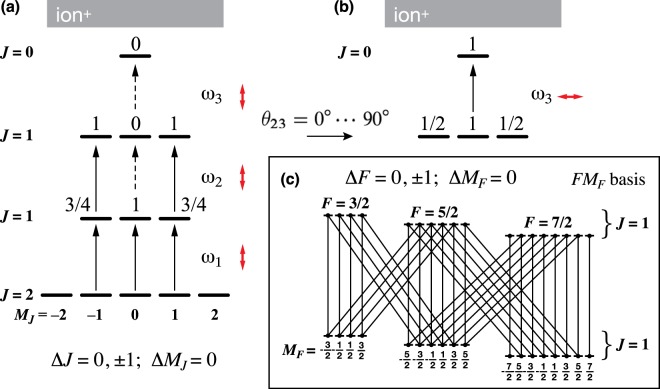
Energy states and transition diagram applicable to (**a**) the even mass isotope, in which the *JM* basis is suitable with a lack of nuclear spin (*I* = 0), and (**b**) changing optical alignment of polarized light causes its selection rules. (**c**) The 1st and 2nd intermediate states described by *FM*_*F*_ basis with non-zero nuclear spin (*I* = 5/2) for the odd mass isotope, showing that cross-linked transitions occur. The red arrows and *ω* indicate linearly polarized light and the transition energy, respectively.

Figure 2 schematically shows the magnetic sublevels (*M*_*J*_) associated with the total angular momentum (*J*) energy states, in which the even mass isotopes are excited via *J* = 2–1–1–0 excitation scheme. The linearly polarized first light (*ω*_1_) quantizes the electronic angular momentum with the electric field axis. Zr atoms have the lowest ground-state of *J* = 2 with five magnetic sublevels *M*_*J*_ = ±2, ±1, and 0. The population for even mass Zr isotopes has an isotropic distribution in each sublevel. With the first light tuned to the 1st excited level, a resonant dipole transition to the *J* = 1 intermediate state occurs. The subsequent intermediate-state populations *P*(*J*′, *M*′), which are prepared by resonant transitions with linearly polarized light, are elaborated using the Wigner–Eckart theorem^17^ by

1 $$P(J{\rm{^{\prime} }},M{\rm{^{\prime} }})\propto \sum _{M{\rm{^{\prime} }}{\rm{^{\prime} }}}{|{d}_{M{\rm{^{\prime} }}M{\rm{^{\prime} }}{\rm{^{\prime} }}}^{J{\rm{^{\prime} }}}(\theta )|}^{2}{(\begin{array}{ccc}J{\rm{^{\prime} }}{\rm{^{\prime} }} & 1 & J{\rm{^{\prime} }}\\ -M{\rm{^{\prime} }}{\rm{^{\prime} }} & 0 & M{\rm{^{\prime} }}\end{array})}^{2}P(J{\rm{^{\prime} }}{\rm{^{\prime} }},M{\rm{^{\prime} }}{\rm{^{\prime} }})$$

where the initial population is denoted by *P*(*J*″, *M*″), the 3*j* symbol represents the relative line strengths, and the reduced rotation matrix elements ${d}_{M^{\prime} M^{\prime\prime} }^{J^{\prime} }(\theta )$ transform the alignment onto the electric-field axis of the probing light. Equation 1 expresses the transition selections of Δ*J* = 0, ±1, and Δ*M*_*J*_ = 0. The resultant population of even mass isotopes in the 1st intermediate state generates the ratios of 3/4, 1, and 3/4 in the magnetic sublevels −1, 0 and +1 magnetic sublevels, respectively.

The second light (*ω*_2_), whose linear polarization is parallel to that of the 1st light (*θ*_12_ = 0°), induces a resonant transition to the 2nd intermediate state (*J* = 1), and the selection rules Δ*M*_*J*_ = 0 and *M* = 0 ↮ 0 are retained because the transition strengths depend on *M*^2^. For the transition from the 1st intermediate state (*J*″ = 1) to the 2nd intermediate state (*J*′ = 1), the populations at the *M*″ = ±1 sublevels in even mass isotopes are projected upward equally. However, the *M*″ = 0 population is excluded. As a result, an aligned state is prepared where the population of the *M*′ = 0 sublevel is zero. In Fig. 2(a), the population of even mass isotopes in the 2nd intermediate state is distributed by a ratio of 1, 0, and 1 for the sublevels *M*_*J*_ = −1, 0, and +1, respectively.

The third light induces a resonant transition to the 3rd intermediate state (*J*′ = 0). Since the 2nd intermediate state (*J*″ = 1) has a zero population and lacks the magnetic sublevel (${M}_{J}^{^{\prime\prime} }=0$), the resulting 3rd intermediate state is unpopulated when linearly polarized light (*ω*_3_) is parallel to the 2nd polarized light (*ω*_2_). Figure 2(b) depicts the case in which the 3rd intermediate state (*J*′ = 0) gains its population by the transition from the 2nd intermediate state (*J*″ = 1) with ${M}_{J}^{^{\prime\prime} }=0$ when polarizations of both types of light are perpendicular to each other (*θ*_23_ = 90°).

Figure 2(c) shows a schematic representation of the |*FM*_*F*_〉 basis, as the coupling *F* = *J* + *I*, in which the sublevels are coupled with the nuclear spin $(I=5/2)$ for the odd mass Zr isotopes. These hyperfine interactions yield a population of odd mass Zr isotopes that are distributed over 18 sublevels in the 1st intermediate state (*J* = 1) and the 2nd intermediate state (*J* = 1). Cross-linkages between the 1st intermediate hyperfine sublevel and the 2nd intermediate hyperfine sublevel render each sublevel accessible. Therefore, the odd mass isotopes do not obey the selection rules dictated in the |*JM*〉 basis but follow Δ*F* = 0, ±1 and Δ*M*_*F*_ = 0 selection rules, which renders the selective photoionization of the odd mass Zr isotopes feasible^13,14^.

To quantify the isotopic selectivity in the selective photoionization scheme for the even/odd mass Zr isotopes, we defined a normalized separation coefficient *β* for odd mass isotope by

2 $${}^{91}\beta =(\frac{\int {I}_{\parallel }{(}^{91}\text{Zr})}{\sum _{m\ne 91}\int {I}_{\parallel }{(}^{m}\text{Zr})})/(\frac{\int {I}_{\perp }{(}^{91}\text{Zr})}{\sum _{m\ne 91}\int {I}_{\perp }{(}^{m}\text{Zr})}),$$

where ${I}_{\parallel }$ and ${I}_{\perp }$ are photoion currents at the parallel polarization (*θ*_23_ = 0°) and perpendicular polarization (*θ*_23_ = 90°), respectively, in a mass spectrum. Since the naturally occurring odd mass isotope of Zr is 91 alone and the remaining isotopes are the even mass (90, 92, 94, 96), we focus on ^91^*β* by Eq. 2 for an evaluation of the odd mass isotopic selectivity. The larger that ^91^*β* becomes, the more selectively photoionized ^91^Zr that is obtained.

## Results

### Four-Step Photoionization Scheme

We have carried out the *J* = 2–1–1–0 excitation scheme, as shown in Fig. 1(a), in the selective odd mass Zr isotope photoionization. Figure 1(b) shows the mass-resolved ^90,91^Zr spectra, frequency-scanning for a 3rd intermediate state, where only the transition for the ^91^Zr isotope was observed, while ^90^Zr (and other even mass isotopes, not shown) was not observed. In the mass spectra measured at 49 551 cm^−1^ with the two different polarizations, as shown in Fig. 1(c), the even mass isotopes were considerably suppressed, while the ^91^Zr isotope remained intact. The maximum resulting separation coefficient ^91^*β* was 2460, which indicates the largest selectivity for the odd mass Zr isotope that has ever been reported^14,16,18^. Green and co-workers^14^ were the first researchers to introduce the *J* = 2–1–1–0 scheme and demonstrated the isotope selectivity (>10) for ^91^Zr. Subsequently, Niki and co-workers^16^ revisited the same scheme and improved as ^91^*β* ~ 70. Both studies utilized the 3rd intermediate state of 52 605.01 cm^−1^. We also revisited the same 3rd intermediate state and substantially obtained ^91^*β* ~ 266 in our setup. This improvement is responsible for rigorous optical alignments in each transition and utilizing a necessary and sufficient laser fluence, which properly reserves the selection rules or alignments. Figure S1 (Supplementary Information) depicts additional details on the ^91^Zr selectivity in various 3rd intermediate states. Table 1 briefly summarizes relevant terms, lifetimes, and cross-sections in a favoured four-step excitation scheme, which is identified in this study. We present detailed aspects of each intermediate state and the ground state.

**Table 1:** The intermediate states associated with selective photoionization of the odd mass Zr isotope.

Intermediate	Energy (cm^−1^)	Term	Cross section (cm^2^)	Lifetime (ns)
1st	17 429.86	$z{}^{3}{{\bf{D}}}_{1}^{^\circ }$	6.7(1) × 10^−15^	<300
2nd	35 046.95	*e* ^5^ **F**_1_	5.1(1) × 10^−14^	7.3 ± 0.5
3rd	49 551.31	(*J* = 0)^a^	3.7(2) × 10^−16^	86.7 ± 1.4

^a^From the angular momentum selection rules that fulfil the selective odd mass Zr isotope photoionization.

The lowest level in the ground Zr is the triplet *a*^3^**F**_2_ with *J*″ = 2. In the heated sample (>2200 K), the neighbouring *a*^3^**F**_3_ (+570.41 cm^−1^) and *a*^3^**F**_4_ (+1240.84 cm^−1^) levels may be thermally populated^19^. However, their contributions are restrictedly excluded by the stepwise photoexcitation. The first intermediate state $z{}^{3}{{\bf{D}}}_{1}^{^\circ }$ was tuned by the 1st laser *ω*_1_ while monitoring its emission to the ground electronic state (${z}^{3}{{\bf{D}}}_{1}^{^\circ }\to {a}^{3}{{\bf{F}}}_{2}$). The lifetime of the 1st intermediate state was estimated by the temporal profile of emission to the ground. Since the typical transition probability between the singlet state and the triplet state is small, the 1st intermediate state (17 429.86 cm^−1^) was long-lived (<300 ns) and consistent with the previously reported data ~243(10) ns^20^. The lifetime of the 2nd intermediate state was obtained by a pump—probe time delay, in which the timing of *ω*_3,4_ pulses with respect to those of *ω*_1,2_ was electronically controlled to change while recording the photoion currents. By fitting a deconvolution procedure to the temporal profile, the lifetime of the 2nd intermediate state *e*^5^**F**_1_ (35 046.95 cm^−1^) was evaluated as 7.3 ± 0.5 ns within the accuracy of the pertinent temporal responses.

As described in the theory of selective photoexcitation, the 2nd step transition ${e}^{5}{{\bf{F}}}_{1}\leftarrow {z}^{3}{{\bf{D}}}_{1}^{^\circ }$ in the *θ*_23_ = 0° alignment is able to render only the odd mass Zr isotope, which is selectively excited by *ω*_2_, as *M*_*J*_ = 0 in *J* = 1 ← 1 transition will not be allowed for the even mass isotopes. Thus, the subsequent excitation by *ω*_3_ to the 3rd intermediate state (*J* = 0 character) is possible only for the odd mass Zr isotope due to its hyperfine sublevel structure. The pump–probe measurement revealed that the 3rd intermediate (49 551.31 cm^−1^) is relatively short-lived with a lifetime of *ca*. 90 ns. The final (or fourth) step is a non-resonant transition to above the ionization potential (I.P. = 53 506.0 cm^−1^) of Zr I^21^, and it was subsequently performed by an intense infrared laser (IR, 1064 nm).

### Optical Alignment of Intermediate State

Optical alignment among the intermediate states is crucial to attain high selectivity. The laser polarization of each step not only is parallel to one another but also requires a high degree of linear polarization. In the optical setup (*vide infra*), the combined 1st and 2nd beams and the 3rd (and fourth IR) beams, either of which was rectified to have a high degree of linear polarizability (>99.99) by a polarizer, are counter-propagating and interact with Zr vapour. The polarization angle between the two combined beams, *θ*_23_, was adjusted by rotations of the polarizer mounted on a motorized rotational stage.

**Figure 3:**
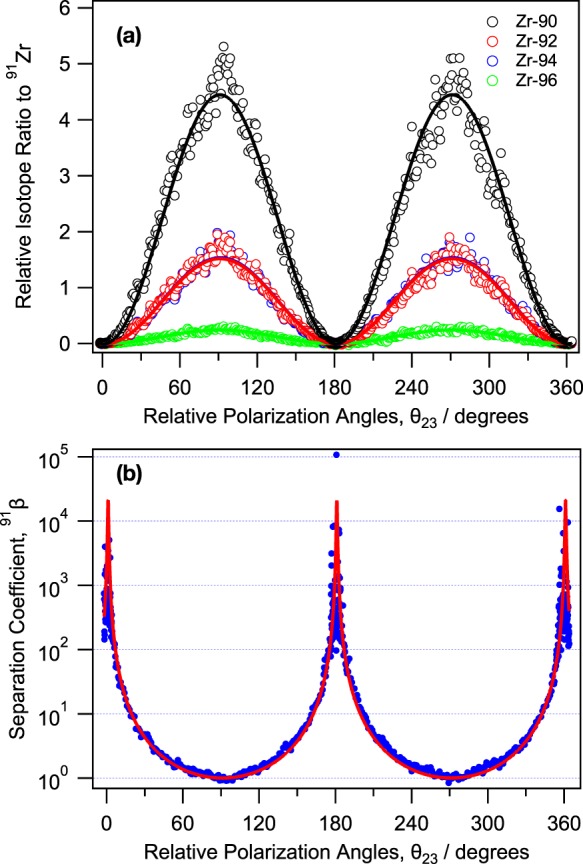
(**a**) Relative isotope ratios to the odd mass isotope ^91^Zr as a function of relative polarization angles of *θ*_23_. Each trace was fitted by ${\sin }^{2}({\theta }_{23})$. (**b**) The polarization angle dependence of the separation coefficient ^91^*β*, calculated from Eq. 2. Both panels use the same 3rd intermediate state of 51 541 cm^−1^, showing that ^91^*β* maximizes in the parallel polarizations.

Figure 3(a) shows the polarization angle dependence of each isotope ratio to ^91^Zr, and each trace was fitted by a sinusoidal form of ${\sin }^{2}({\theta }_{23})$. At *θ*_23_ = 0, 180, and 360° ^91^Zr isotope is selectively photoionized, whereas the other even mass isotopes (^90^Zr, etc.) were suppressed nearly to zero. At the angles of 90 and 270°, the appearing ^90,92,94,96^Zr ratios to ^91^Zr attain 4.45(2), 1.52(1), 1.54(1), and 0.242(2), respectively, which are in accordance with the natural abundance ratios (4.585, 1.528, 1.549, and 0.250, respectively)^22^. This finding implies that our experimental setup is free from an undesirable saturation effect that may compromise the odd mass selectivity predicted from the selection rules. In Fig. 3(b), ^91^*β* as a function of the relative polarization angle of *θ*_23_ shows a sharp response in a narrow range, where the selectivity drastically changes on 3–4 orders of magnitude. For this reason, we repeatedly checked the polarization alignment during the search for favoured intermediate states or a reliable evaluation of *β*.

### Absorption Cross Section for Intermediate State

**Figure 4:**
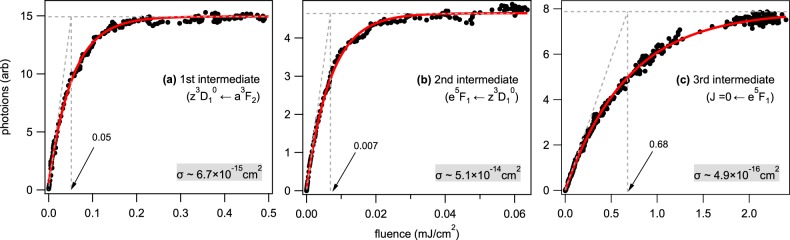
Responses of photoions vs input laser fluence in the *J* = 2–1–1–0 scheme: (**a**) The first excited-state intermediate (${z}^{3}{{\bf{D}}}_{1}^{^\circ }\leftarrow {a}^{3}{{\bf{F}}}_{2}$), (**b**) the second excited-state intermediate (${e}^{5}{{\bf{F}}}_{1}\leftarrow {z}^{3}{{\bf{D}}}_{1}^{^\circ }$), and (**c**) the third excited-state intermediate (*J*′ = 0 ← *e*^5^**F**_1_). The black dots and red curves depict the observed and the fitted, respectively, by Eq. 3, and the dashed lines denote its asymptotes and tangents at each zero fluence. The arrows indicate the nominal saturation fluence, and the obtained cross-sections *σ* are listed in Table 2.

Figure 4 shows the response profiles of photoions vs. input fluence in each intermediate-state transition employed in the stepwise *J* = 2–1–1–0 excitation. Each panel depicts (a) the first intermediate state (${z}^{3}{{\bf{D}}}_{1}^{^\circ }\leftarrow {a}^{3}{{\bf{F}}}_{2}$), (b) the second intermediate (${e}^{5}{{\bf{F}}}_{1}\leftarrow {z}^{3}{{\bf{D}}}_{1}^{^\circ }$), and (c) the third intermediate (*J*′ = 0 ← *e*^5^**F**_1_) at the energy of 51 848 cm^−1^. We observed either of the ^90^Zr or ^91^Zr ions and ensured that no differences in their behaviours had to be considered. The fluence inputs from the other lasers, which are not associated with the probing transition, were increased but did not produce ions. We have varied the fluence by a round continuously variable neutral density filter, which was controlled by a stepper motor. Absorption cross-sections were determined by fitting the observed response profiles by Eq. 3 from a simple rate-equation model^14,18^. The intensity of the populating ions is

3 $$I/{I}_{\text{s}}=1-\exp (\,-\frac{\sigma F}{h\nu }),$$

where *I*_*s*_ is an asymptotic intensity in *I* that reaches a plateau as an input laser fluence *F* (J/cm^2^) is increased. The cross section *σ* of the intermediate state can be obtained as a fitting parameter. A nominal saturation fluence, where the maximum photoion intensity *I* is 1 − 1/e of *I*_*s*_ is also expressed with *F*_s_ ≡ *hν*/*σ*. The dashed lines in these panels denote its asymptotes and tangents at zero fluence. The arrows indicate the saturation fluence, which is obtained by the fit (or with a graphical analysis); they show agreement with the nominal *F*_s_, as shown in each panel of Fig. 4.

From the saturation measurements, the 1st intermediate state has the nominal fluence of 50 *μ*J/cm^2^, whereas the 3rd intermediate is less likely saturated at 0.68 mJ/cm^2^. The 2nd intermediate is most likely saturated as a small fluence of 7 *μ*J/cm^2^. The saturation fluence *F*_*s*_ is inversely proportional to the cross section, which is tabulated in Table 1. Note that the 2nd intermediate state has a crucial role, in which the transition to this state facilitates the isotopic selection based on the optical alignment and the angular momentum selection rules are critically imposed. Therefore, a careful adjustment of the *ω*_2_ fluence is required to ensure that the selection rules are not impaired. The 2nd intermediate state is larger *σ* ~ 5 × 10^−14^ than the 1st (~7 × 10^−15^) or the 3rd (~5 × 10^−16^ cm^2^) intermediate states. A short lifetime (7 ns) of the 2nd level indicates large absorption and high oscillator strength, which enhances the transition strength in favour of the excitation by a comparable temporal width of the laser. The 3rd intermediate state is the smallest state *σ*, and other 3rd intermediate states (*vide infra*) indicate a similar order of magnitude, 10^−16^ cm^2^. Table 2 lists cross-sections of these 3rd intermediate states (Supplementary Information, Fig. S3). Page *et al*.^18^ measured the cross-section for autoionizing Rydberg states beyond the I.P. limit of Zr I. Our values are consistent with their reports.

**Table 2:** Comparison of isotopic selectivity, relative ion yield, lifetime, and cross-section for various third intermediate states employed in the selective photoionization of the odd mass zirconium isotope.

3rd intermediate state (cm^−1^)	^91^ *β*	relative yield		lifetime (ns)	cross-section (cm^2^)
		^91^Zr	${{\boldsymbol{\sum }}}^{{\bf{2}}{\bf{m}}}\,{{\bf{Zr}}}^{{\bf{a}}}$		
***J*** ** = 2–1–1–0 + IR**					
52 605.01^b^	266	1	1	1450(50)	1.8(2) × 10^−16^
	~70^c^	—	—	—	—
	>10^d^	—	—	—	—
52 343.66	310	3.8	3.3	174(5)	≪2.7(2) × 10^−19^
51 848.17	575	30	<13.8	182(5)	4.9(2) × 10^−16^
51 801.65	490	4.2	3.6	885(20)	4.2(2) × 10^−16^
51 154.00	1480	16.6	<3.0	110(2)	1.2(2) × 10^−16^
49 551.31	2460	17.1	1.8	86.7(14)	3.7(2) × 10^−16^
49 136.64	1630	17.2	2.8	450(10)	1.7(7) ×10^−15^
***J*** ** = 2–1–1–0**					
54 413.0	9.2	5.5	160		

Note that one standard deviation as statistical errors are reported in the parentheses.

^a^The total even mass zirconium observed in TOF mass spectra.

^b^The previously reported intermediate state^14,16^, used for benchmarks of Zr-91 isotope.

selectivity and ionization efficiency in this study. ^c^Evaluated from data reported by Niki *et al*.^16^.

^d^From Green *et al*.^14^.

### Search for Potential Third Intermediate States

**Figure 5:**
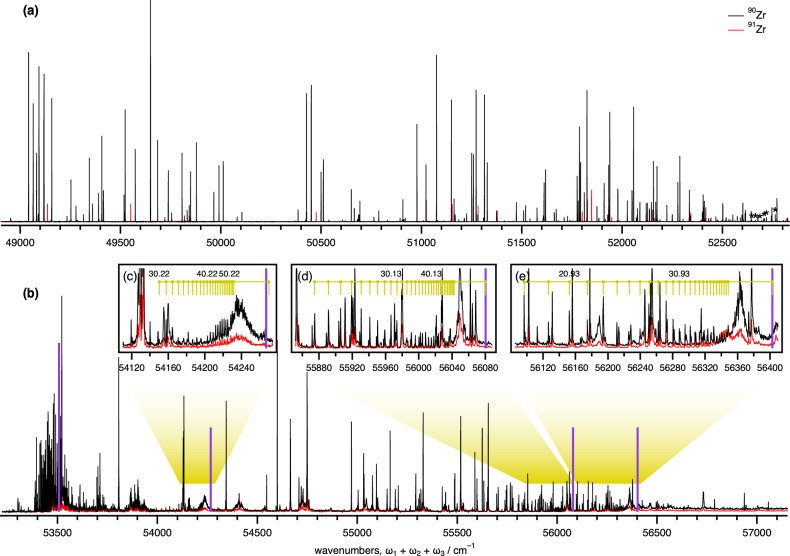
(**a**) The mass-resolved photoionization spectra of ^90,91^Zr probes the potential 3rd intermediate states by a scan of *ω*_3_ via *J* = 2–1–1–0 scheme. Both spectra for ^90^Zr (black) and ^91^Zr (red) appear mostly overlapped; the ^91^Zr states that show selectivity are distinctive by comparison. The peaks marked by asterisks denote multiphoton processes other than the desired scheme. (**b**) The same spectra but explored the vicinity and beyond the first ionization potential of Zr I. Note that the relative intensities in the panel (b) are scaled down by 25 with respect to (a). The inset panels (**c**–**e**) are the expanded portions of the spectra (b), where the Rydberg series converge to the Zr II ion’s second (54 270.3, +763 cm^−1^), 4th (56 079.9, +2572 cm^−1^), and 5th (56 402.9, +2895 cm^−1^) excited states, as indicated by each purple stick (unscaled).

As the study progressed, we have identified several favoured 3rd intermediate states that show not only a higher degree of the isotope selectivity but also a higher photoionization efficiency. Figure 5(a) shows the mass-resolved photoionization spectra for ^90,91^Zr, which probes potential 3rd intermediate states with the odd-mass selectivity. The frequency scans by *ω*_3_ were performed downward from the previously recorded 52 605 cm^−1^ region. The laser fluences of *ω*_1_, *ω*_2_, and *ω*_4_ (IR) were maintained at mostly the same levels across a wide range (~4000 cm^−1^), which was covered by the use of seven different kinds of laser dyes. The odd-mass selective ion signals identify themselves as the selective ^91^Zr compared to either ^90^Zr spectra or the other even-mass isotopes (not shown). Table 2 lists the discovered 3rd intermediate states and relevant properties, such as *β*, ion yields, lifetimes, and cross sections.

In addition to the 3rd intermediate state (49 551.3 cm^−1^) shown in Fig. 1(c) that indicates the highest ^91^*β* ~ 2460, other 3rd states observed in the low-energy region yield significantly large *β* values. The relative ion yields of the ^91^Zr isotope were evaluated from simple comparisons of the benchmarked ion yield at the 52 605.0 cm^−1^ state. The 51 848.17 cm^−1^ state produces a maximum of 30× photoions, and its selectivity remains modest (^91^*β* ~ 575). The relative yields for the even-mass isotopes should be taken into account when the selective odd mass photoexcitation is applied. Thus, we explicitly elaborate the even-mass isotope contaminations in the fourth column of Table 2. The lifetimes for each intermediate state were obtained by temporal profiles measured in the pump—probe measurements, as shown in Fig. S2 (Supplementary Information). Note that an isotopic enrichment of ^91^Zr may be expressed by its fraction of the observed total ions, which is indicative of >99.7% for the case of 49 551.3 cm^−1^, which shows *β* ~ 2460. Nevertheless, the *β* (Eq. 2) has a built-in advantage to assess a total isotopic selectivity; the intermediate-state alignments with light polarization matter in the stepwise photoexcitation for the laser isotope separation.

We further extended the search above the 1st I.P. of Zr I, *viz*., autoionizing levels in Zr II, which can be accessed by three photons without the fourth IR photon. Since the odd mass isotope selective condition is being held during probing, the terminal state would show some selectivity if an autoionizing state held the *J* = 0 character. Figure 5(b) displays a whole view for the mass-resolved photoionization spectra of ^90,91^Zr, exploring the vicinity and beyond the first I.P., where the ion yields indicated a dramatic (over one order of magnitude) increase and gradually faded for the high energy while the background continuum became apparent. We have discovered a 54 413 cm^−1^ level of energy that shows minimal isotopic selectivity, ^91^*β* ~ 9, as shown in Fig. S5 (Supplementary Information). Despite the high total ionization efficiency, the ^91^Zr photoions do not gain as much yield as expected. Consequently, the yields of even mass isotopes are not suppressed and remain relatively high (160×, Table 2).

### High-lying Rydberg Series and Ionization Potential in Zr I

In the aforementioned excitation scheme, the 3rd laser *ω*_3_ tunes near the first I.P. limit in Zr I. These high-lying Rydberg states are probed and even ionized immediately after absorbing the photon in a static electric field (without absorbing the fourth IR photon). To avoid level structures perturbed by the preexisting electric field, we employed a delayed, pulsed ion collection that utilizes a pulsed electric field and delays after the last photon impinges and subsequently induces field-ionization of a Rydberg atom.

**Figure 6:**
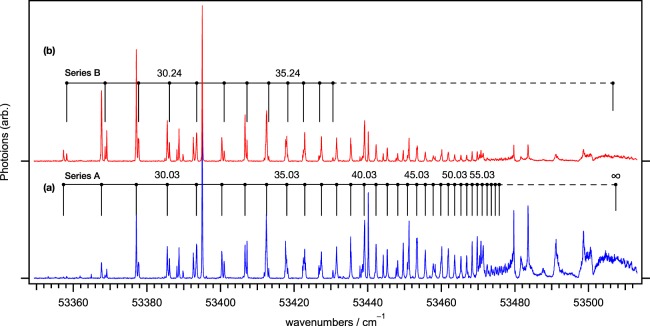
The delayed, field-ionized Rydberg series of Zr-90, expanded in the vicinity of I.P. limit from Fig. 5(b), were obtained by the following configurations: (**a**) the normal gating and (**b**) time-delayed gating to observe metastable or slowly ejecting Rydberg atoms. Series A and B are assigned and converge to the I.P. limit of Zr I (53 507.4(3) cm^−1^). The leaders denote the effective principle number n^*^ for each series, and the mark of ${n}^{\ast }\sim \infty$ depicts an I.P. limit obtained by the fit.

Figure 6 shows expanded views of the delayed, field-ionized Rydberg series in Fig. 5(b). Towards the red-side of the Rydberg series, an ion extraction was seemingly delayed as observed arrival times in time-of-flight (TOF) in Fig. S4 (Supplementary Information). A Rydberg atom, which is close-lying at the ionization threshold with the imposed electric field, may undergo metastable behaviour. Both traces depict the same spectra, with the exception of their intensities; slow-arrived ions are observed in the upper states (b), while prompt ions in TOF signals are captured in the lower states (a). Note that a Rydberg atom with a larger principle quantum number *n* is known to be relatively long-lived compared to a smaller *n*; their radiative lifetime is proportional to *n*^3^ in the finite temperature condition^23^. Although we are unable to explain why lower *n* states that reversely appear to be more intense, the trace (b) supplements the frequencies of weaker states, which are barely detectable in (a). We identified 33 lines, which span ~120 cm^−1^ from approximately 53 357 cm^−1^ to the 1st ionization limit. Although the spectral patterns or features appear complex and irregular, as typically seen in any Rydberg series, we extracted two series (A and B) due to the periodicity of the Rydberg series, which likely has the same convergence limit.

To analyse the series, we have applied the standard formula

4 $$\mathop{\nu }\limits^{ \sim }={\mathop{\nu }\limits^{ \sim }}_{{\rm{\infty }}}-\frac{{R}_{\text{m}}}{(n-\mu {)}^{2}},$$

which describes the Rydberg series observed in our data. Here, ${\tilde{{\rm{\nu }}}}_{\infty }$ corresponds to the I.P. limit of Zr I, *R*_m_ is the mass-corrected Rydberg constant for ^90^Zr, 109 736.75 cm^−1^, and *μ* is the quantum defect, which is defined by *μ* = *n* − n^*^, where n^*^ denotes the effective quantum number. The least squares fit to the observed series A with Eq. 4 gives ${\tilde{{\rm{\nu }}}}_{\infty }$ and *μ* as 53 507.4(3) cm^−1^ and 0.03(1), respectively. Series A and B seemingly consist of a doublet that converges to the same I.P. limit, which may be attributed to some perturbation or coupling with Rydberg states that may reside nearby, none of which are convincingly addressed in this study. A table that lists the wavenumber of each peak assigned to the Rydberg series is available in the Supplementary Information (Table S1).

Hackett *et al*.^21^ also observed and assigned Rydberg levels of n^*^ > 50, which appear at higher-lying levels in a narrow window (~20 cm^−1^). Recently, Hasegawa *et al*.^24^ have revisited the Rydberg series via various intermediate states and measured the lower-lying series (n^*^ ~ 23–39) that span a wide window of ~200 cm^−1^. Our observation of Rydberg series have similarly undergone the low-lying levels in the transition from the higher intermediate state *e*^5^**F**_1_ (35 046.95 cm^−1^). The value (53 507.4(3) cm^−1^) obtained for the 1st ionization potential of ^90^Zr I, which converges to the singly ionized Zr II ($4{d}^{2}5{s}^{4}{{\bf{F}}}_{\mathrm{3/2}}$), shows agreement with the previously reported values, *viz*. 53 506.0(3)^21^ and 53 507.8(5)^24^ cm^−1^.

### Autoionizing Rydberg Series in Zr II

Above the I.P. limit of Zr I^25^ (>53 507 cm^−1^), the spectrum became congested and exhibit a continuum towards the blue spectrum (higher energy). Several levels stem from the singly ionized Zr II and fall into the spectral range that we observed in Fig. 5(b). Via the 2nd intermediate *e*^5^**F**_1_ state (35,046.95 cm^−1^) in the *J* = 2–1–1 scheme, we observed a moderately intense Rydberg series that converges to the Zr II ion’s 2nd (54 270.3 cm^−1^), 4th (56 079.9 cm^−1^), and 5th (56 402.9 cm^−1^) excited states, as shown in the inset three panels (c–e) in Fig. 5. Each of these states correspond to the energy levels of singly ionized zirconium (Zr II)^19,25^: $4{d}^{2}5s{a}^{4}{{\bf{F}}}_{\mathrm{7/2}}$ (+763 cm^−1^), $4{d}^{3}{b}^{4}{{\bf{F}}}_{\mathrm{3/2}}$ (+2572 cm^−1^), and $4{d}^{3}{b}^{4}{{\bf{F}}}_{\mathrm{5/2}}$ (+2895 cm^−1^), respectively. Our analyses to these Rydberg states were undertaken in the same way for the Rydberg series of Zr I (*vide supra*), and the reduced energy levels for Zr II are approximately consistent with Moore^25^ and Hasegawa^24^. However, our primary intent was not to drive a full identification on the Zr II electronic structure.

The series (n^*^ ~ 30–50 with quantum defect near 0.22) converges to the +763 cm^−1^ level in Fig. 5(c) produces ${\tilde{{\rm{\nu }}}}_{\infty }=54\,270.3(1)$ cm^−1^. So-obtained I.P. for the 2nd level 4*d*^2^5*sa*^4^***F***_7/2_ (+763 cm^−1^) is consistent with 54 269.4(3)^19,25^, but is larger by *ca*. 3 cm^−1^ than 54 267^24^ cm^−1^. The limits determined for the 4th excited state and 5th excited state are 56 079.9(2), and 56 402.9(3) cm^−1^, respectively, and are consistent with the reported values^19^. A table that lists the wavenumber of each peak assigned to the Rydberg series is available in Supplementary Information (Table S2).

Most of the spectral lines observed in the Zr II Rydberg series were not specifically broad compared with the Zr I Rydberg series. A typical linewidth was ≈0.5 cm^−1^ (FWHM) by a Gaussian/Lorentzian fit to the series, as measured at +763, + 2572, and +2895 cm^−1^, whereas the typical linewidth of <0.2 cm^−1^ (FWHM) for the Zr I series was consistent with all combined laser bandwidths. This linewidth of the Zr II Rydberg series may identify an upper limit of the lifetime of these states, which is estimated to be ≈10 ps. Thus, autoionizing Rydberg levels are short-lived.

## Discussion

In the laser even/odd mass isotopic separation, angular momentum selection rules that are pertinent to linearly polarized light and hyperfine interactions are important to facilitate odd mass isotopic selectivity. Selectively photoionizing odd mass Zr isotopes are sequentially irradiated by three resonant lasers to raise the energy from the ground state (*J* = 2), to a 1st intermediate state (*J* = 1), to a 2nd intermediate state (*J* = 1), and to a 3rd intermediate state (*J* = 0) prior to photoionization by a non-resonant laser pulse (*J* = 2–1–1–0 scheme). Throughout the comprehensive search for suitable 3rd intermediate states (Fig. 5), which need the *J*′ = 0 character, we have identified several favoured intermediate states that produce high odd mass selectivity and relatively high ion-yield efficiency. These states indicate that the odd mass isotopic selectivity is located in the relatively lower energy region (49 000–52 000 cm^−1^), and the spectral feature is sparse and displays a cutoff of approximately 49 000 cm^−1^. Among these 3rd intermediate states, the 51 848.17 cm^−1^ shows ^91^*β* ~ 575 and produces approximately 30-fold more photoions compared with that of the 52 605.01 cm^−1^ state^14,16^. Other states to note are 51 154.00 cm^−1^ (*β* ~ 1480, 17×), 49 551.31 cm^−1^ (*β* ~ 2460, 17×), and 49 136.64 cm^−1^ (*β* ~ 1630, 17×), as shown in Table 2. A high degree of isotopic selectivity or *β* is attained, e.g., ~10^3^, which is even obtained in the AVLIS process, where narrow-band lasers are precisely tuned to the frequency of a targeted hyperfine transition to selectively excite and ionize an isotope. Among the discovered intermediate states, a maximum ^91^Zr isotope enrichment of >99.7 is attained. In this favourable outcome, triple resonant photoexcitation can be a strategy for enriching isotopes.

Optical alignment of intermediate states also has a critical role in isotope separation to ensure maximum selectivity. The method uses the selection rules that are applicable to linearly polarized light to prepare each intermediate state, including magnetic sublevels, where the even mass isotopes are unpopulated. However, hyperfine interactions the population isotropically distribute in the magnetic sublevels for the odd mass isotopes. The odd mass isotopes are excited from the intermediate states and are photoionized. The desired outcome is that all three linearly polarized lasers are perfectly parallel and need a high degree of polarization. This result can be achieved by employing prism polarizers. We carried out counter-propagating alignments between the combined 1st and 2nd beams and the 3rd (and fourth IR) beam, either polarization of which was rectified by passing a polarizer. The polarization that matched between both arms was then controlled by adjusting a motorized rotation stage. In this manner, switching between a parallel configuration and perpendicular configuration was swiftly and accurately performed for reliable evaluation of *β*.

Another aspect to take into account is the amount of input laser fluence pertinent to each step transition. For instance, high-intensity laser fields may redistribute requisite sublevel populations in the even mass isotope and subsequently ruin selectivity. Nonetheless, we may require a certain amount of fluence inputs that efficiently gain the photoions. Hence, the absorption cross-section of each transition is a crucial parameter to be elucidated. From the data presented in Fig. 4, typical cross-sections (cm^2^) for the 1st, 2nd, and 3rd step transitions were estimated on the order of −15, −14, and −16, respectively. These cross-sections correspond, respectively, to 50 *μ*J/cm^2^, 7 *μ*J/cm^2^, and 0.7 mJ/cm^2^ as the nominal saturation fluence for the odd mass isotope selective ionization, which is also shown in Fig. S3 (Supplementary Information). In addition, the 2nd intermediate state has a larger cross-section than others and primarily holds the selective excitations, which are indicative of the essential transition step in the entire sequence. We have employed a higher fluence from each nominal fluence in the measurements, where any negative effects against the selectivity, such as an increase in the ion currents, were observed (Fig. S3). Thus, even the saturation effect is positive in our strategy.

We generated atomic vapour in a hostile environment equipped with an electron beam bombarding, which may be a potential cause of interfering electric and magnetic fields that cause redistribution of the sublevel populations and destroy angular momentum selection rules. These formidable influences on selectivity were not detected, probably due to relatively small hyperfine splittings of Zr atoms^11^. As aforementioned, Doppler line broadening, which is associated with the vapourization temperature (>2200 K), does not diminish the selective photoexcitation, since our laser bandwidths (≤8 GHz) thoroughly address broadened, sparsely hyperfine-split transitions. Doppler line broadening favourably operates and thus gains compared with the AVLIS, which usually needs to minimize line broadening in narrow-band laser excitations.

Reducing the number of lasers utilized for the even/odd-mass separation would be extremely desirable in terms of system complexity, cost, and maintenance. Studies that utilize two-colour multi-photon photoionization in the ALVIS process^12^ and the even/odd-mass selection rules^26,27^ were reported. However, schemes based on the selected levels appear to be less promising for selectivities due to a poor hyperfine structure or high fluence, which smears selective transitions. Accordingly, it was a primary reason to extend the search to autoionizing Rydberg states, which are accessed only by three photons in the *J* = 2–1–1–0 scheme (without carrying the fourth IR photon). Knowledge of the Zr II metastable level energies enable the identification of portions of the autoionizing Rydberg series, which potentially possess a distinct advantage to the ionization efficiency in the terminal state. If this autoionizing state showed a significant advancement in the isotopic selectivity, *viz*., with a pure *J* = 0 character, it would be the most favoured state to be undertaken. Regardless of our attempt, however, we were not able to identify potential candidates of autoionizing Rydberg series that suitably implements the three-step photoionization. Amongst the notable autoionizing states that were searched, the level of 54 413 cm^−1^ barely indicates an isotopic selectivity of ^91^*β* ~ 9 and a lower ionization efficiency compared with those carried in the *J* = 2–1–1–0 + IR scheme (Table 2). This result may be ascribed to the Rydberg mixing in the backbone continuum of singly ionization state^18^, which produces a large cross-section with a relatively short lifetime of sub-nanoseconds.

Our results highlight that rationalizing odd-mass isotope selective photoionization by multiple lasers is profoundly beneficial in pre-treatment prior to processing nuclear transmutation by acceleration colliders at a beam factory. Our study elaborates the theory and methods in the selective photoionization scheme, which are applicable to high-level radioactive odd mass isotopes, such as ^93^Zr and ^95^Zr, that are fission products with long lifetimes generated as by-products in nuclear plants. Without detailed knowledge of hyperfine energy level structures for the odd mass radioactive isotopes, the discovered 3rd intermediate states should be thoroughly adapted to facilitate laser isotope separation.

Three-photon resonant, stepwise photoionization in zirconium vapour is a very useful and efficient technique for selecting odd mass isotopes. However, alternatively suitable autoionizing Rydberg states, which potentially enhance the ionization efficiency and isotopic selectivity, have not been detected by searching to 58 000 cm^−1^. These observations may indicate that zirconium has a more complex level structure than closed shell atoms, such as palladium^15^. In the *J* = 2–1–1–0 excitation scheme, however, we have identified several third intermediate excited states, which show both significant isotopic selectivity and high ionization efficiency for the odd mass Zr isotope. Fundamental knowledge, such as obtained cross section, lifetime, and separation coefficient information, are crucial in scaling up multistep laser excitation schemes for isotope separation of atomic zirconium. We believe that the parameters reported in this paper would suffice for a surmountable scheme towards a primary application to laser separation of radioactive waste.

## Methods

### Experimental Setup

**Figure 7:**
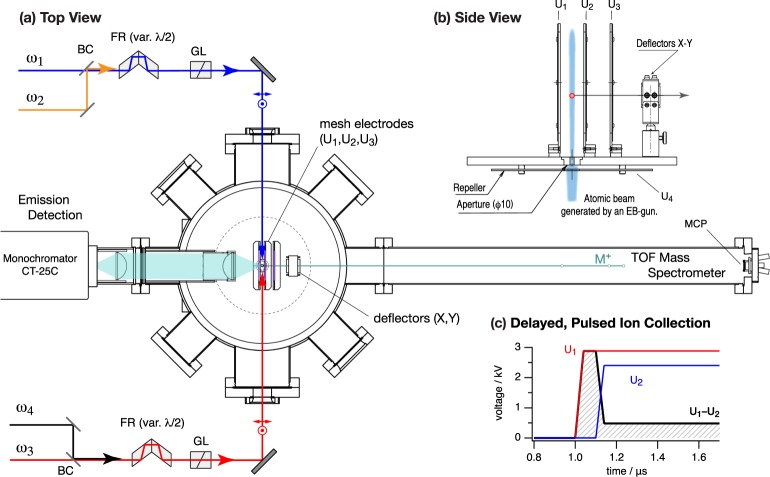
Schematic setup: (**a**) A source vacuum chamber and a Wiley–McLaren-type TOF mass-spectrometer; the two arms, combined with two of the four beams by beam combiners (BC), in given polarization configurations rectified by double-type Fresnel rhombs (FR) and Glan-Laser (GL) polarizers are counter-propagated and overlapped at the centre, where three electric wire meshes are placed. (**b**) A skimmed atomic beam generated by an EB-gun intersects at a right angle with the laser beams is extracted to the right upon photoionization. (**c**) The timing used for a delayed, pulsed ion collection, which is necessary to explore low-lying Rydberg states. The effective electric fields follow the voltage sequence (black solid) that extracts cations (*U*_3_ is always set to zero).

An electron beam (EB) gun (EGK-3/HPS-510S, ULVAC Technologies Inc.) was employed to vapourize Zr atoms that are collimated and then interact with subsequent three/four laser irradiations (bandwidths 6–8 GHz, duration <10 ns, and repetition rate 10 Hz) for the resonant multi-step photoionization. We typically employed three dye lasers, which were pumped by two separate Nd:YAG lasers (second or third harmonics pumping), and a fourth pulse, if used, that carried small fraction of the YAG fundamental (1.06 *μ*m) from a residue of harmonic mixings. The two beams (*ω*_1_ and *ω*_2_) were collinearly combined with a 50:50 beam splitter. The other beams (*ω*_3_ and *ω*_4_) were counter-propagated to a vacuum chamber, as shown in Fig. 7(a). All four beams were linearly polarized with all electric vectors parallel. For both combined beams, passing through Glan-Laser (GL10-A, Thorlabs Inc.) prism polarizers rectifies the degree of their linear polarizations (the extinction ratio >100 000). To establish the perpendicular polarization configuration, a pair of double Fresnel rhombs (FR; OptoSigma) were used to orthogonally rotate the polarization of either arm. The timing sequence of the four laser pulses was controlled either optically or electrically to the pump lasers; typical sequence: *ca*. 15 ns for *ω*_2_ to *ω*_1_ pulse, and less than a few ns for *ω*_3_ and/or *ω*_4_ to *ω*_2_. The input pulses were partly overlapped in time, the sequence of which is a result of the optimization of gaining the maximum photoions. We have meticulously checked any possibility for the non-resonant multi-photon ionization processes other than the desired schemes, which are not significantly observed during the scanning.

Photoions were perpendicularly accelerated via a set of three electrode grids, and the cations were detected as a function of the arrival time at a microchannel plate (MCP; Hamamatsu Photonics, F4655-11), which consists of a Wiley–McLaren-type TOF mass spectrometer (≈1 m long; mass resolution *m*/Δ*m* > 600), Fig. 7(b). So-obtained photocurrents were fed to a digital oscilloscope (Tektronix, MDO3054, 2.5 GS/s) for signal processes with a multiple-gated integration associated with each mass-channel of interest. Mass-selective photoionization spectra of Zr isotopes were recorded as a function of the sum of the excitation energy (*ω*_1_ + *ω*_2_ + *ω*_3_) by scanning either of the laser wavelengths. In probing the high-lying Rydberg states near the I.P. limit, we employed a pulsed, field ionization method that utilizes a delayed high-voltage applied to the grids and induces the field ionization, which is advantageous to the investigation of low-lying excited states that are unperturbed by any static electric fields. The electric fields were delayed to apply (1 *μ*s) upon laser irradiation, in which a pulsed electric field was initially introduced for a short duration (<100 ns, ~820 V/cm) and then switched to a soft electric field (<200 V/cm), as illustrated in Fig. 7(c). The instant and high electric field effectively perturbs the low-lying Rydberg states and induces field ionization. So-generated ions are then accelerated by the two-step electric fields in the three grids that give the convergence in the flight times and record a mass-resolved spectrum of each isotope.

### Samples and Light Sources

A sample of Zr shots (99.9%, 3–5 mm*ϕ*) was applied as received (Rare Metallic Co.), and its isotopic composition was checked via non-resonant photoionization signals of ZrO^+^, which was reasonably matched to the natural abundance of Zr^22^. A carbon hearth linear (2.6 cm^3^) was installed into a crucible, where the EB-gun bombards and ionizes Zr atoms, which helps to deduce intermittent and unwanted discharges around the sample source and often causes an electric interference or extensive damage to the electronic devices in the experiment. We used two Lumonics (HD-500) and a Sirah (Credo) tunable dye lasers and mostly scanned *ω*_3_ to probe the 3rd excited intermediate states using a wide variety of laser dyes (Rhodamine 6 G, Pyrromethene 597, mixed Rhodamine B + Rhodamine 101, DCM, Pyridine 1, and Styryl 8) for the four steps of photoionization incorporated with an IR. For the three-photon photoionization, shorter wavelengths dyes such as Coumarin 102, Coumarin 47, Coumarin 480, Coumarin 503, and Coumarin 540 A were used to probe autoionizing Rydberg states beyond the ionization potential of Zr I. The employed laser wavelengths were monitored by a wavelength meter (Coherent, WaveMaster, accuracy ± 0.005 nm) at all times. The measured signal intensities for all photoionization spectra were normalized to its input laser intensity, which was monitored by a photodiode.

### Data Analyses

The lifetime of the intermediate states was determined by either emission detected with DC-level-based decay curves or a photoion profile as a function of the pump–probe delay time of the two pump lasers, which are electronically controlled to scan by a digital delay/pulse generator (Stanford, DG535). So-measured decay profiles were fitted with deconvolution by nonlinear least square procedures. The fluence dependence of the photoions to each intermediate level was evaluated by varying the relevant laser powers with a round continuously variable neutral density filter (Thorlabs), whose angles were PC-controlled and programmed to make stepwise rotations by a stepper motor stage (OptoSigma). A beam profiler (Ophir/Spiricon, SP928) selected a 1/*e*^2^ intensity that served as a beam diameter associated with the fluence conversion, and the total input power was measured by a pyroelectric energy sensor (Thorlabs, ES111C).

## Supplementary information

Supplemental Information
