# Supplementary material for: Selective Resonance Photoionization of Odd Mass Zirconium Isotopes Towards Efficient Separation of Radioactive Waste
Source: Sci Rep. 2019 Feb 11;9:1754. doi: 10.1038/s41598-018-38423-4 (PMC6370825; doi:10.1038/s41598-018-38423-4)
Supplement: Supplementary file 1 — Supplemental Information [file 41598_2018_38423_MOESM1_ESM.pdf]

Supplementary Information

Selective Resonance Photoionization of Odd Mass Zirconium Isotopes Towards Efficient Separation of Radioactive Waste

Takashige Fujiwara,[1, a)] Tohru Kobayashi,[1] and Katsumi Midorikawa[1]
Attosecond Science Research Team, RIKEN Center for Advanced Photonics, 2-1 Hirosawa, Wako, Saitama 351-0198 Japan.

[a)]Electronic mail: t.fujiwara@riken.jp.

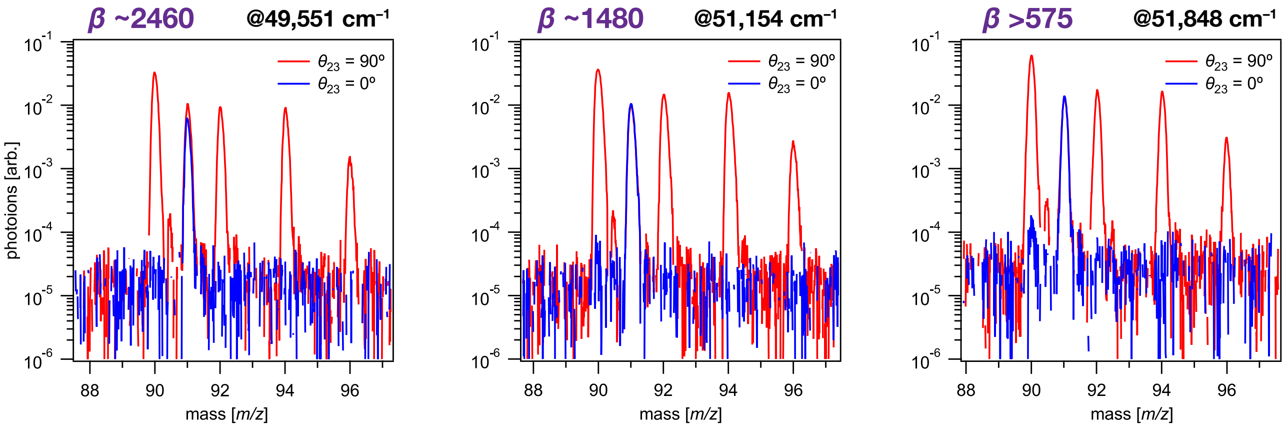

Figure S1. The higher isotopic selectivity were observed in low-lying 3rd-excited intermediate states, rather than those in the higher intermediates at the present J=2–1–1–0 excitation scheme. The mass resolution in our TOF-MS is estimated to be >600. Note that the signals of even-mass isotopes, especially for @49,551 cm^{-1} buried under the noise levels, are beyond our detection limit.

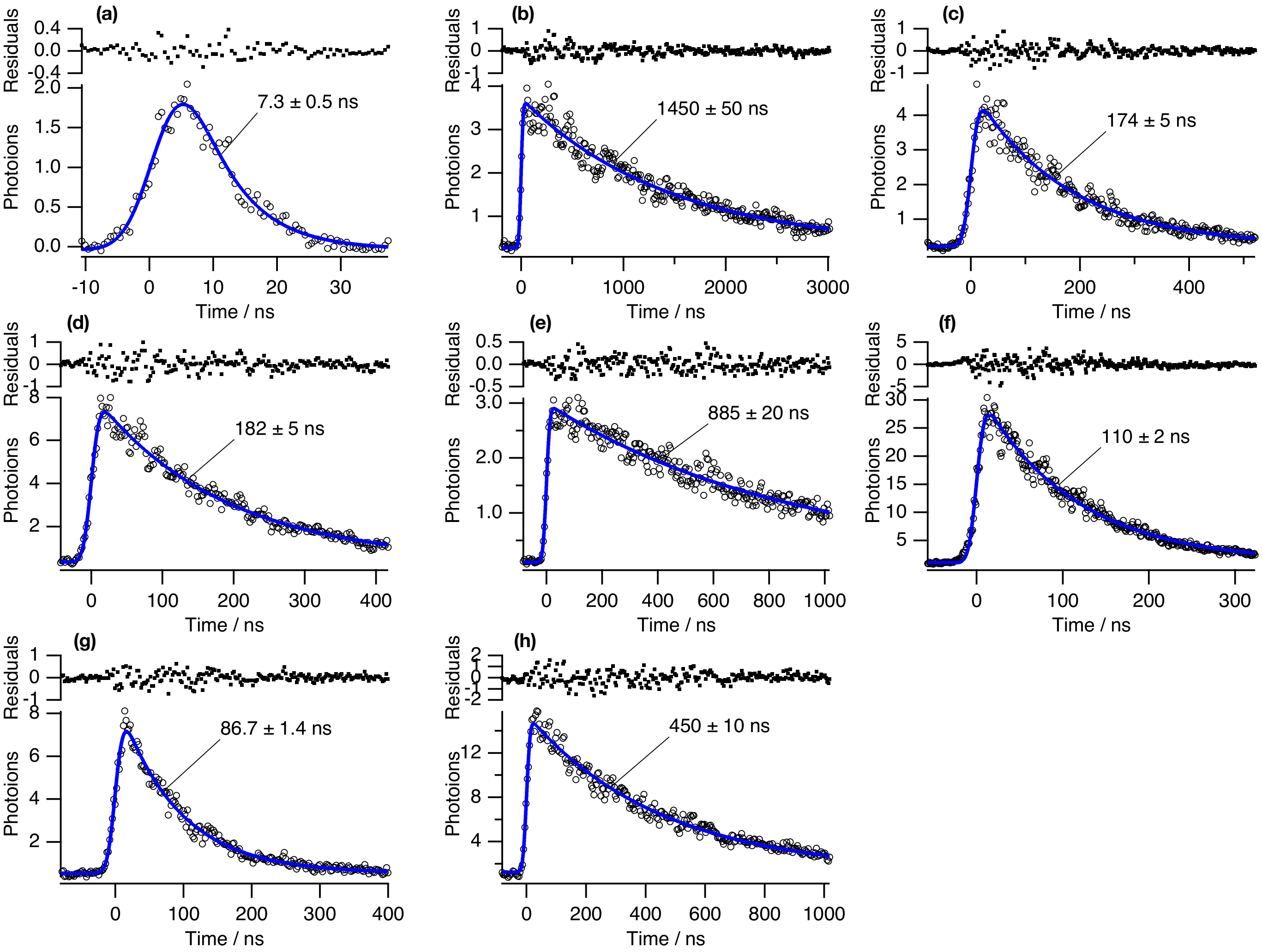

Figure S2. The temporal profiles of photoions of various intermediate states investigated in this study using the pump–probe delay techniques. (a) The 2nd intermediate (35 046.95 cm^{-1}) probed in the four step photoionization. The lifetime of the probed state was evaluated by the nonlinear least-square fit with a deconvolution procedure, and accompanied by the accuracy of a standard deviation (1σ). The rest of profiles depict probing 3rd intermediate states: (b) 52 605.01, (c) 52 342.66, (d) 51 848.17, (e) 51 801.65, (f) 51 154.00, (g) 49 551.30, and (h) 49 136.64 cm^{-1}.

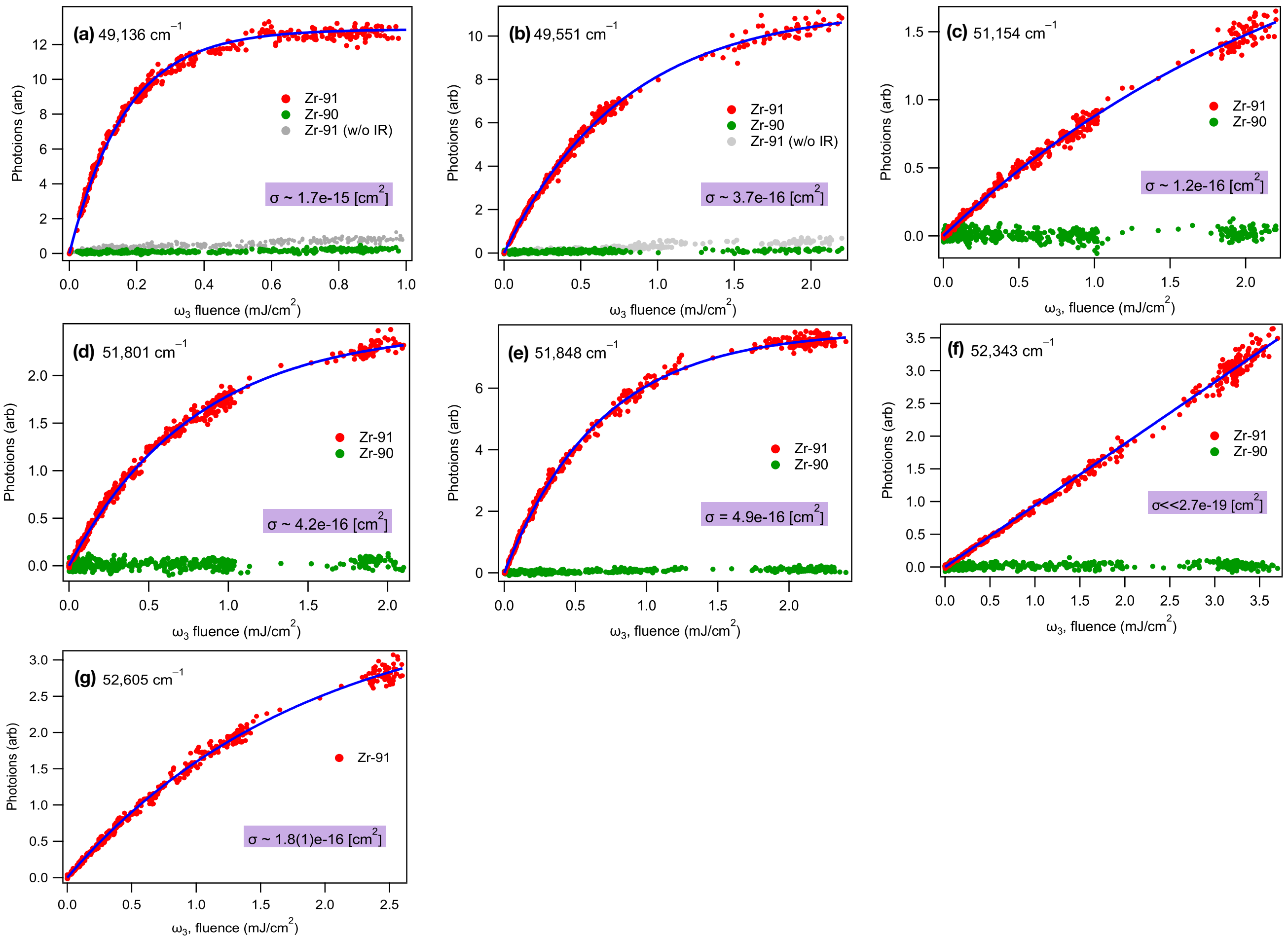

Figure S3. Saturation curves of photoions vs. laser fluence of ω_3 for various third-excited intermediates in the J=2–1–1–0 scheme. Each panel depicts the ^{91}Zr isotope selective photoionization (red dots) with its fit curve (blue) by Eq. 3; ^{90}Zr photoions (green dots) were simultaneously obtained, and^{91}Zr (gray dots) were also measured without the presence of the fourth IR photons. The resultant absorption cross sections σ were estimated by the fit. Note that most of the curves shown reach a plateau as fluence increasing; for 52 343 cm^{-1} level, its response seemingly remains linear, indicating a significantly small cross section.

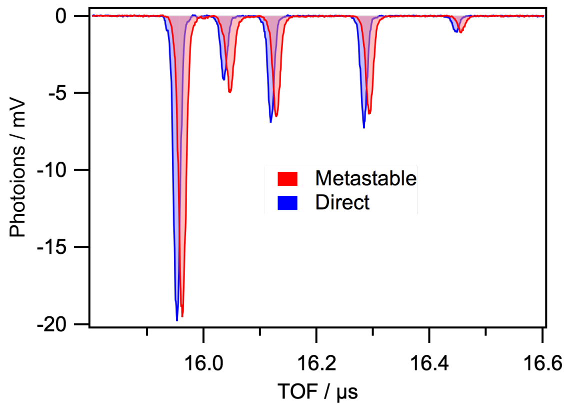

Figure S4. Time-of-flight (TOF) mass-selective photoions of Zr, obtained in the vicinity of the ionization limit (Zr I), where metastable Rydberg stats are delayed-ionized by the pulsed field ionization (red trace), compared to direct photoionization (blue). An advantageous collecting of those ions was carried out via setting time-delayed gates with respect to those directed TOF sions in order to closely reveal low-lying Rydberg series (Refer to Figure 7).

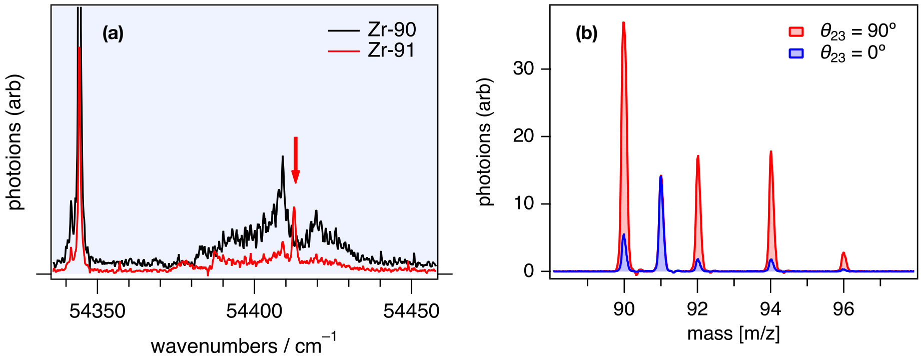

Figure S5. (a) The three-steps photoionization spectra of ^{90,91}Zr acquired in the odd-mass isotope selective condition ($\theta_{23} = 0°$). The red arrow points the 54 413.0 cm^{-1} state (Table 2 and Figure 5(b)), which partially indicates the selective ^{91}Zr ionization due to the state holding a $J = 0$ character. (b) Time-of-flight (TOF) mass spectra, measured at 54 413.0 cm^{-1} state with the two different optical alignments ($\theta_{23} = 0°$ and 90°), consequently yields $^{91}\beta \sim 9.2$. The other even-mass isotopes are modestly suppressed and unfavorably leave a significant contribution to the total photoions.

Table S1. Rydberg series energy (±0.01 cm^{-1}) and effective quantum number (n^*) of transitions in Zr I atom from the high-lying intermediate $e\,^5\mathbf{F}_1$ state (35,046.95 cm^{-1}).

Series A		Series B	
n^*	Ry level (cm^{-1})	n^*	Ry level (cm^{-1})
27.03	53357.34	27.24	53358.22
28.03	53367.67	28.24	53368.64
29.03	53377.13	29.24	53377.72
30.03	53385.55	30.24	53386.15
31.03	53393.44	31.24	53393.55
32.03	53400.36	32.24	53400.99
33.03	53406.65	33.24	53407.21
34.03	53412.50	34.24	53413.10
35.03	53418.01	35.24	53418.29
36.03	53422.90	36.24	53422.57
37.03	53427.46	37.24	53426.94
38.03	53431.54	38.24	53430.55
39.03	53435.39		
40.03	53439.04		
41.03	53442.24		
42.03	53445.32		
43.03	53448.17		
44.03	53450.83		
45.03	53453.32		
46.03	53455.66		
47.03	53457.80		
48.03	53459.88		
49.03	53461.88		
50.03	53463.62		
51.03	53465.32		
52.03	53466.91		
53.03	53468.38		
54.03	53469.78		
55.03	53471.12		
56.03	53472.45		
57.03	53473.58		
58.03	53474.66		
59.03	53475.75		

Table S2. Rydberg series energy (±0.01 cm^{-1}) and effective quantum number (n^*) of transitions in singly ionized Zr II atom from the high-lying intermediate $e\,^5\mathbf{F}_1$ state (35,046.95 cm^{-1}). The converging Rydberg levels are relative to the I.P limit of a neutral Zr I atom (53 507.4 cm^{-1}).

$4d^2 5s\ a\ ^4\mathbf{F}_{7/2}$ (+763 cm^{-1})		$4d^3\ b\ ^4\mathbf{F}_{3/2}$ (+2572 cm^{-1})		$4d^3\ b\ ^4\mathbf{F}_{5/2}$ (+2895 cm^{-1})	
n^*	Ryd level (cm^{-1})	n^*	Ryd level (cm^{-1})	n^*	Ryd level (cm^{-1})
30.22	54150.20	23.13	55874.88	18.93	56096.68
31.22	54157.79	24.13	55891.60	19.93	56126.54
32.22	54164.40	25.13	55905.86	20.93	56152.33
33.22	54170.82	26.13	55919.24	21.93	56174.53
34.22	54176.54	27.13	55930.84	22.93	56194.07
35.22	54181.81	28.13	55941.12	23.93	56210.95
36.22	54186.63	29.13	55950.37	24.93	56226.27
37.22	54191.32	30.13	55959.26	25.93	56239.87
38.22	54195.57	31.13	55966.61	26.93	56251.59
39.22	54199.08	32.13	55973.61	27.93	56262.28
40.22	54202.75	33.13	55980.43	28.93	56271.81
41.22	54205.98	34.13	55986.20	29.93	56280.44
42.22	54208.92	35.13	55991.11	30.93	56288.19
43.22	54211.70	36.13	55995.84	31.93	56295.22
44.22	54214.35	37.13	56000.22	32.93	56301.71
45.22	54216.69	38.13	56004.25	33.93	56307.50
46.22	54218.89	39.13	56008.12	34.93	56312.91
47.22	54220.96	40.13	56011.80	35.93	56317.87
48.22	54223.01	41.13	56015.13	36.93	56322.50
49.22	54224.92	42.13	56018.12	37.93	56326.78
50.22	54226.80	43.13	56021.10	38.93	56330.44
51.22	54228.30	44.13	56023.57	39.93	56334.25
52.22	54229.91	45.13	56025.85	40.93	56337.50
53.22	54231.64	46.13	56028.14	41.93	56340.22
		47.13	56030.43	42.93	56343.19
		48.13	56032.54	43.93	56346.16
		49.13	56034.48	44.93	56348.36
		50.13	56036.22		
		51.13	56037.98		
		52.13	56039.58		
		53.13	56041.16		
		54.13	56042.39		
		55.13	56043.97		
